# Cells in somatosensory areas show synchrony with beta oscillations in monkey motor cortex

**DOI:** 10.1111/j.1460-9568.2007.05890.x

**Published:** 2007-11

**Authors:** Claire L Witham, Minyan Wang, Stuart N Baker

**Affiliations:** Institute of Neuroscience, Newcastle University Sir James Spence Building, Royal Victoria Infirmary, Queen Victoria Road, Newcastle upon Tyne NE1 4LP, UK

**Keywords:** coherence, parietal, sensorimotor, single unit

## Abstract

Oscillatory synchronization between somatosensory and motor cortex has previously been reported using field potential recordings, but interpretation of such results can be confounded by volume conduction. We examined coherence between single-unit discharge in somatosensory/parietal areas and local field potential from the same area as the unit, or from the motor cortex, in two macaque monkeys trained to perform a finger movement task. There were clear coherence peaks at ∼17.5 Hz for cells in the primary somatosensory cortex (both proprioceptive and cutaneous areas) and posterior parietal cortex (area 5). The size of coherence in all areas was comparable to previous reports analysing motor cortical cells and M1 field potentials. Many coherence phases clustered around –π/2 radians, indicating zero lag synchronization of parietal cells with M1 oscillatory activity. These results indicate that cells in somatosensory and parietal areas have information about the presence of oscillations in the motor system. Such oscillatory coupling across the central sulcus may play an important role in sensorimotor integration of both proprioceptive and cutaneous signals.

## Introduction

Beta-band oscillations in humans and monkeys occur in both the primary motor (M1) and somatosensory (S1) cortex, as well as the posterior parietal cortex (Murthy & Fetz, 1992; Salmelin & Hari, 1994; MacKay & Mendonca, 1995; Witham & Baker, 2007). Recent findings suggest that oscillations play a role in sensorimotor processing (Riddle & Baker, 2005; Baker *et al.*, 2006; Witham & Baker, 2007).

Previous studies have shown coherence between local field potentials (LFPs) from the parietal cortex and M1 (see Murthy & Fetz, 1992; Brovelli *et al.*, 2004). However, field potentials spread by volume conduction. This is probably limited in recordings from penetrating microelectrodes, but no quantitative data are available. Volume conduction would artefactually increase coherence between two areas, as well as invalidating assessment of phase differences, as electrical cross-talk has near zero phase lag.

Single-unit recordings are spatially highly localized, as neural spikes can only be recorded from cells close to the electrode tip. However, assessment of synchronization using paired single-unit recordings is difficult, as non-linearities in the spiking process greatly attenuate coherence values (see Baker *et al.*, 2003). The true incidence of coherence is thus probably underestimated due to statistical thresholding.

A useful alternative method is to calculate coherence between single units and LFP (Fries *et al.*, 2002; Baker *et al.*, 2003; Soteropoulos & Baker, 2006). This combines the selectivity of single units with the sensitivity of population activity. It should be relatively immune to artefactual effects, for example caused by common noise, as the frequency bands used to record spikes and LFP are non-overlapping. With this method, Soteropoulos & Baker (2006) successfully demonstrated coupling between the cerebellum and M1. A similar approach has been applied to the peripheral nervous system (Christakos, 1994).

Whilst in non-invasive studies the postcentral cortex is treated as a single area, S1 in fact comprises distinct cytoarchitectonic areas with different functions (Nelson *et al.*, 1980). Areas 3a and 2 receive mainly proprioceptive inputs (Nelson *et al.*, 1980); area 3a additionally probably makes corticomotoneuronal connections to gamma motoneurons (Rathelot & Strick, 2006), giving it direct control of spindle feedback gain. By contrast, areas 3b and 1 receive predominantly tactile information, with differences in receptive field size and specificity (Nelson *et al.*, 1980). Moving further posterior, area 5 has proprioceptive-related cells (Mountcastle *et al.*, 1975). Baker *et al.* (2006) showed that oscillatory afferent feedback was present in muscle spindle afferents, but absent in putative cutaneous afferents. It is therefore interesting to determine whether only proprioceptive areas of S1 show coherence with M1 oscillations, or whether the phenomenon is more widespread. Additionally, given the known differences even between areas with similar proprioceptive vs cutaneous preferences, examining coherence with M1 by specific cortical area may produce new insights into the functional role of this activity.

In this study, we examined coherence across the central sulcus using single-unit recordings from distinct S1 or parietal areas, and simultaneous measurements of M1 LFP. We show robust oscillatory synchrony between M1 and S1/area 5 cells, agreeing with a role for oscillations in sensorimotor integration.

## Materials and methods

### Behavioural task

Two female rhesus macaques (*M. mulatta*) performed an index finger flexion task for food reward (monkeys M and L). The finger was inserted into a narrow tube, which splinted the finger and constrained movement to the metacarpo-phalangeal joint. The tube was mounted on a lever, which rotated on an axis aligned to this joint. Lever movement was sensed by an optical encoder, and a motor exerted torque in a direction to oppose flexion. This was programmed to act like a spring (initial torque 48 mNm). The task required movement into target (between 6 ° and 24 ° flexion) and holding for 2 s (torque required at target either 64 mNm or 128 mNm). Motor torque then rose, and the animal released the lever to obtain its reward. The analysis reported here focuses on the hold period, as this has previously been shown to contain the strongest beta-band activity (Baker *et al.*, 1997, 2001; Witham & Baker, 2007).

### Surgical preparation

Following behavioural training, each monkey was implanted under general anaesthesia and aseptic conditions with a headpiece (to allow head fixation) and a recording chamber placed over the central sulcus (Lemon, 1984; Baker *et al.*, 1999). The anaesthesia consisted of 3.0–5.0% sevoflurane inhalation in 100% O_2_ supplemented with a continuous infusion of intravenous alfentanil (0.025 mg/kg/h). A full program of postoperative analgesia (10 µg/kg buprenorphine; Vetergesic; Reckitt and Colman Products, 5 mg/kg carprofen; Rimadyl; Pfizer) and antibiotic care (10 mg/kg cefalexin; Ceporex; Schering-Plough Animal Health or 15 mg/kg amoxycillin; Clamoxyl LA; Pfizer) followed surgery. All procedures were carried out under the authority of licences issued by the UK Home Office under the Animals (Scientific Procedures) Act 1986, and in accordance with the European Communities Council Directive of 24 November 1986 (86/609/EEC).

### Recording

In daily experiments, a 16-channel microdrive (Eckhorn & Thomas, 1993), loaded with microelectrodes or tetrodes, was used to record single-unit activity and LFPs from M1, somatosensory and parietal areas. The different cortical areas were identified by a clinical examination of unit receptive fields and by noting the motor responses to intracortical microstimulation (13–18 biphasic pulses, 300 Hz, 0.2 ms per pulse, currents up to 50 µA). After recordings from M1 were complete, three microwire electrodes (50 µm diameter stainless steel wire insulated with Teflon, AM790500, A-M Systems, Carlsborg, WA, USA; tip impedance ∼30 kΩ at 1 kHz) were implanted transdurally in M1, and fixed in place with cyanoacrylate glue and dental cement. These electrodes were positioned in rostral M1 on the precentral gyrus, with tips 2–3 mm apart. This permitted recording of M1 LFP simultaneously with single units from other areas. Spike waveforms (300 Hz−10 kHz bandpass) were sampled continuously at 25 kHz, and saved to hard disc together with LFPs sampled from the same electrodes at 500 Hz (1–100 Hz bandpass), lever position, M1 LFPs (bandpass 1–100 Hz, sampling rate 500 Hz, inverting amplifier, negativity upwards) and task behavioural markers. Spike occurrence times were discriminated offline using custom-written cluster cutting software (Getspike, S.N. Baker). Only clean single units with consistent wave shapes and no interspike intervals <1 ms were used for subsequent analysis.

### Analysis

Coherence was estimated using LFP recordings sampled at 500 Hz; unit spike trains were converted to a waveform sampled at 500 Hz (by counting the number of spikes in 2-ms bins, Baker *et al.*, 2003). Coherence was calculated between single units and M1 LFPs, and also between single units and local (same area) LFPs. For single unit to M1 LFP coherence, the LFPs from the different microwires in M1 were averaged together before use to yield a low-noise representation of M1 activity. For single unit to local LFP coherence, all simultaneously recorded local LFPs (excluding the LFP recorded from the same electrode as the single unit) were averaged.

Four non-overlapping sections each 256 sample points long were extracted from each task hold period, and processed using a Fast Fourier Transform, giving a frequency resolution of 1.95 Hz. Coherence was calculated using formulae given in (Baker *et al.*, 2006). Coherence was considered significantly different from zero (*P* < 0.05) if it was greater than *Y*, where (1)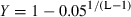 and *L* is the total number of non-overlapping sections (Rosenberg *et al.*, 1989). Coherence magnitude was averaged across all cells in a given population; significance limits were assigned to these averaged coherence spectra as described in Evans & Baker (2003). *Z*-scores were used to compare unit to local LFP coherence with unit to M1 LFP coherence, and were calculated as follows: (2)
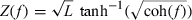
where coh(*f*) is the coherence at frequency *f*. For comparison, the *Z*-scores were summed over the frequency bins of interest (*f*_1_ to *f*_2_) and bias-corrected by subtracting the *Z*-scores over an equal number of frequency bins (*n*_*f*_) in a frequency range of no interest (*g*_1_ to *g*_2_, in the 200–230 Hz range), as follows: (3)
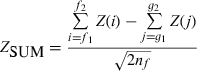
Coherence phase was calculated as the argument of the cross-spectrum. Confidence limits on the phase were calculated as: (4)
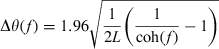
For some cells, phase was linearly related to frequency, indicating a fixed time delay. Phase delays were calculated by fitting a line to the phase–frequency plot using linear regression and calculating the slope. Delays are presented as the maximum likelihood value returned by the regression fit and the 95% confidence interval. For each cell the mean phase was found by taking the circular mean of phases over a given frequency range. The population mean phase in a given cortical area (denoted by 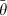) was found by taking the circular mean of the individual mean phases (θ_*n*_), according to Fisher (1993), as: (5)
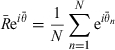
where *N* is the total number of cells, and 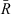
is the ‘mean resultant length’. 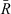
provides a measure of the consistency of phase across a population. It varies from 0 to 1, where 0 is complete cancellation of phases and 1 is no cancellation (phases are identical). Confidence limits for the population mean phase were also calculated for all samples of a suitable size, using the methods detailed in Fisher (1993). The Raleigh test for uniformity with unspecified mean direction was used to test whether 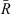
was significantly different to zero.

For the M1 LFPs (recorded using either microelectrodes acutely inserted with the Eckhorn drive, or with chronic microwires), absolute power spectra were calculated using the method detailed in Witham & Baker (2007). Time-resolved power spectra were also calculated using the wavelet-based method detailed in Baker & Baker (2003). All analysis routines were implemented in the MATLAB package (The MathWorks, Natick, MA, USA).

### Histology

At the end of experiments, monkeys were deeply anaesthetized (pentobarbitone, 60 mg/kg i.p.) and perfused through the heart with phosphate-buffered saline (pH 7.2) followed by 4% formal saline fixative. For both monkeys, 50-µm sagittal sections of the sensorimotor cortex were cut and stained with Cresyl violet. These were used to confirm the location of the different cortical areas.

## Results

A total of 174 cells were recorded from area 3a (109 from monkey M and 65 from monkey L), 98 cells were recorded from area 2 (90 from M and eight from L) and 120 cells were recorded from area 5 (44 from M and 76 from L). Although the primary focus of this work was to record from somatosensory areas with deep receptive fields, a small dataset was also obtained in monkey L from area 3b (six cells) and area 1 (15 cells). Each neuron was present for at least 50 trials (providing >200 non-overlapping sections for the coherence analysis).

The ability of cells to carry oscillations in their discharge is dependent on their firing rates (Baker *et al.*, 2003). The mean firing rates for the cells in this analysis were comparable to those found in M1 cells in the same animals (a more extensive analysis of firing rate for these cells can be found in Witham & Baker, 2007). Monkey M had mean firing rates of 13.6 ± 1.2 Hz for M1 (102 cells), 17.3 ± 1.6 Hz for area 3a, 12.7 ± 1.5 Hz for area 2 and 19.2 ± 2.1 Hz for area 5 (mean ± standard error). Monkey L had firing rates of 14.1 ± 0.9 Hz for M1 (169 cells), 21.3 ± 3.9 Hz for area 3a, 9.3 ± 2.9 Hz for area 2 and 9.7 ± 1.5 Hz for area 5. At these rates, single-unit coherence with beta-band oscillations is possible, although it is likely to be at a low level (Baker *et al.*, 2003; Soteropoulos & Baker, 2006).

### Single-cell examples

**Fig. 1:**
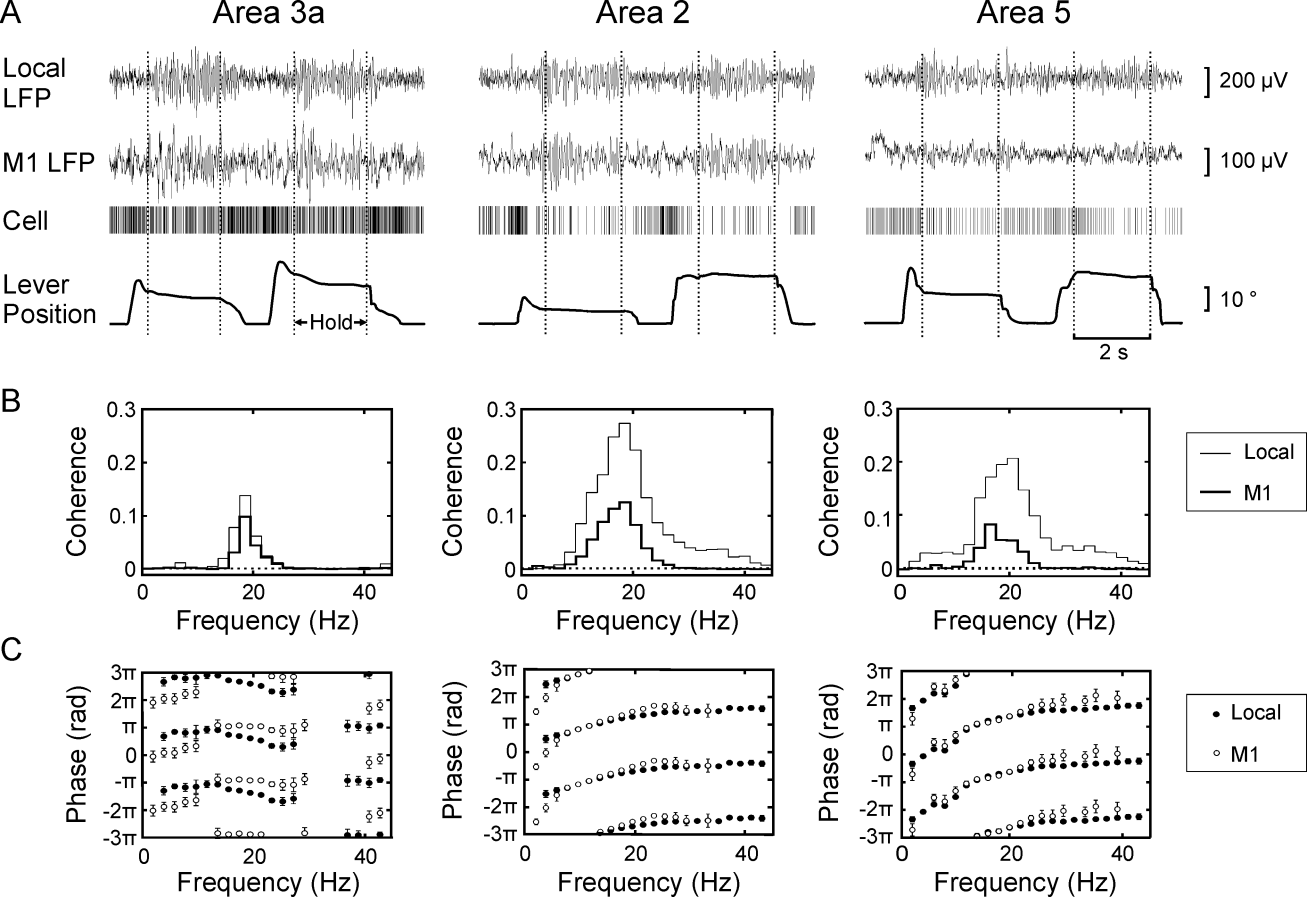
Examples of cells recorded from area 3a, area 2 and area 5. (A) Raw traces of local LFP, M1 LFP and lever position for two consecutive trials, together with spike times of a simultaneously recorded cell. The vertical dotted lines indicate the start and end of the hold period. (B) Unit to local LFP (thin line) and unit to M1 LFP (thick line) coherence spectra for each of the three cells. The dotted line shows significance level (*P* < 0.05). (C) Unit to local LFP (filled circles) and unit to M1 LFP (open circles) phase spectra for each of the three cells. Each phase point has been plotted three times separated by 2π to avoid wrap-around effects when fitting regression lines. LFP, local field potential; M1, primary motor cortex.

Figure 1 shows example raw data for cells recorded from area 3a, area 2 and area 5 in monkey L. Local LFP, M1 LFP and lever position traces, together with spike times, are shown in Fig. 1A. The unit to local LFP coherence (thin line) and unit to M1 LFP coherence (thick line) are shown in Fig. 1B. All three cells showed peaks in their coherence spectra in the beta frequency range (∼20 Hz), well above the significance level (dotted line). The coherence phases are illustrated in Fig. 1C for both unit to local LFP coherence (filled circles) and unit to M1 LFP coherence (open circles). For both the area 2 and area 5 cells, the phase–frequency relationship was similar for local or M1 LFP, with a linearly increasing phase across the beta frequency range. The positive slope indicates that the cells lead the LFP. However, for both cells the implied phase delays (calculated for the 13.7–23.4 Hz range) were longer for unit to M1 LFP coherence (29.9 ± 1.3 ms for the area 2 cell and 31.4 ± 9.2 ms for the area 5 cell) than for unit to local LFP coherence (17.4 ± 3.6 ms for the area 2 cell and 21.2 ± 4.7 ms for the area 5 cell). The area 3a cell had different phase–frequency relationships for unit to local LFP coherence (linearly decreasing across the beta frequency range; phase delay of 26.4 ± 7.7 ms) and unit to M1 LFP coherence (constant phase across the beta frequency range; slope not significantly different to zero, *P* > 0.05, regression analysis).

### Coherence amplitude

**Fig. 2:**
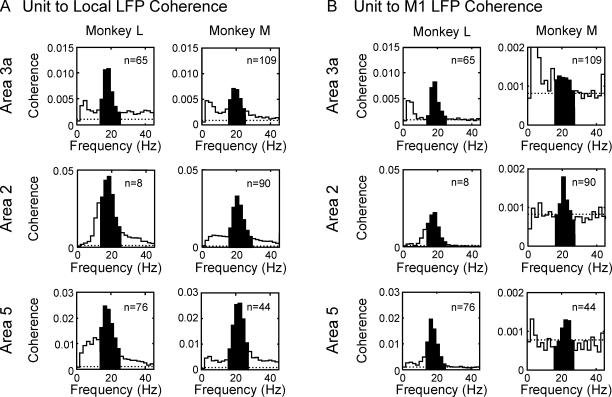
Average coherence spectra for each monkey and area. (A) Unit to local LFP coherence. (B) Unit to M1 LFP coherence. The dotted lines show the significance level (*P* < 0.05). The shaded areas show the frequency range used in *Z*-score and phase analysis. LFP, local field potential; M1, primary motor cortex.

The average coherence spectra for each monkey and area are shown in Fig. 2 for unit to local LFP coherence (Fig. 2A) and unit to M1 LFP coherence (Fig. 2B). For both monkeys there were clear peaks in the unit to local LFP coherence in the beta frequency range. These peaks were well above the significance level in all cases. There were also significant peaks in the unit to M1 LFP coherence for both animals and across all areas. However, the size of coherence was much smaller for monkey M than monkey L. The unit to M1 LFP coherence peak frequencies for monkeys L and M, respectively, were: area 3a, 17.6 Hz for both monkeys; area 2, 17.6 Hz and 19.5 Hz; area 5, 15.6 Hz and 21.5 Hz (the unit to local LFP coherence peak frequencies were identical).

Cells were considered to have significant beta-band coherence if at least two frequency bins were above the significance limit between 15.6 Hz and 25.4 Hz for monkey M, and between 13.7 Hz and 23.4 Hz for monkey L. This criterion was chosen from the binomial probability distribution to yield an overall significance level of *P* < 0.05. The proportions of cells with significant coherence were almost identical between the two monkeys for unit to local LFP coherence in each of the three areas. In area 3a, 57.0% of the cells (37/65) from monkey L had significant coherence compared with 56.0% of cells (61/109) from monkey M. Area 2 had the largest proportion of significant cells, with 87.5% of the cells (7/8) from monkey L and 86.6% of the cells (78/90) from monkey M having significant coherence. Finally, in area 5, 75.0% of cells (57/76) from monkey L and 77.3% of cells (34/44) from monkey M had significant coherence. However, for unit to M1 LFP coherence the proportions of significant cells were considerably higher in monkey L (42/65 cells in area 3a; 6/8 cells in area 2; 51/76 cells in area 5) than in monkey M (26/109 cells in area 3a; 28/90 cells in area 2; 14/44 cells in area 5).

**Fig. 3:**
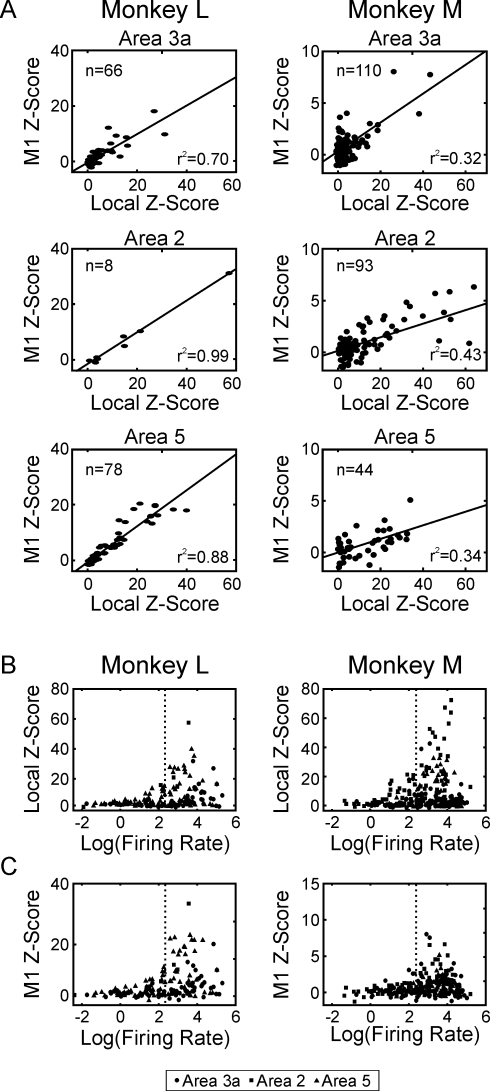
Correlation plots. (A) Unit to local LFP *Z*-scores vs unit to M1 LFP *Z*-scores for each monkey and cortical area. The line shows best fit from regression analysis. (B) Log of mean firing rate vs unit to local LFP *Z*-scores. (C) Log of mean firing rate vs unit to M1 LFP *Z*-scores. The dotted line in (B) and (C) represents 10 Hz firing rate used to separate high and low rate cells in the analysis described in the text. M1, primary motor cortex.

We compared the unit to local LFP and unit to M1 LFP coherence values across the units by measuring the summed *Z*-transformed coherence [*Z*-score, see Materials and methods, Eq. (3)] over the frequency range of interest (shaded areas in Fig. 2). Figure 3A shows the correlation between the unit to local LFP *Z*-scores and the unit to M1 LFP *Z*-scores (referred to as local *Z*-scores and M1 *Z*-scores, respectively). For both animals and all areas there were clear significant linear relationships between the local and M1 *Z*-scores (*P* < 0.05, regression analysis; *r*^2^ values are shown on the plots). However, the slopes obtained from the best-fit lines for monkey L (0.52 ± 0.04 for area 3a, 0.59 ± 0.07 for area 2 and 0.66 ± 0.06 for area 5) were larger than those obtained for monkey M (0.14 ± 0.04 for area 3a, 0.06 ± 0.02 for area 2 and 0.07 ± 0.03 for area 5).

The level of coherence is known to be dependent on the firing rate of the cell. We investigated the correlation between the *Z*-scores (both for unit to local LFP coherence and for unit to M1 LFP coherence) and the cells' mean firing rate during the task hold period. The results are shown in Fig. 3B for unit to local LFP *Z*-scores, and Fig. 3C for unit to M1 LFP *Z*-scores. Data from each monkey are shown individually, but the results from cortical areas are overlain. In all cases, low coherence values were seen at all firing rates, whereas high coherence occurred only for cells with higher rates.

For all four plots there was a significant linear relationship between *Z*-score and rate (regression analysis, *P* < 0.05). However, the *r*^2^ values were low (0.14 for local LFP coherence and 0.15 for M1 LFP coherence in monkey L, 0.04 for local LFP coherence and 0.05 for M1 LFP coherence in monkey M), reflecting the high scatter in the plots. We also looked at the difference in mean *Z*-score for firing rates below and above 10 Hz (dotted line on plots). For monkey L, the mean unit to local LFP *Z*-scores were significantly different when separated by rate in this way (3.41 ± 0.57 for rates below 10 Hz; 13.33 ± 1.79 for rates above 10 Hz, *n* = 73, 76 cells, respectively; mean ± standard error of mean; *P* < 0.001, *t*-test). Similar results were found for all four plots in Fig. 3B and C.

### Coherence phase

**Fig. 4:**
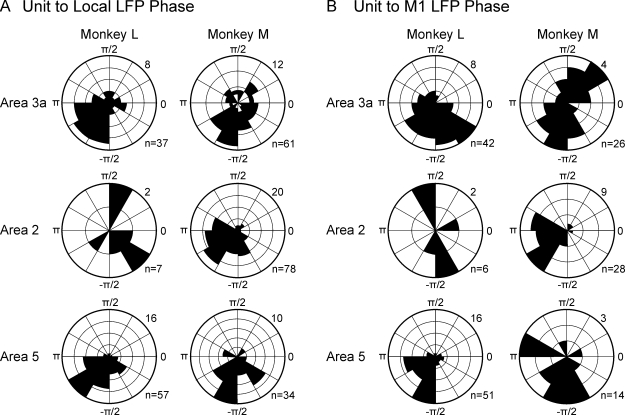
Phase histograms for each monkey and area. (A) Unit to local LFP coherence phase histograms. (B) Unit to M1 LFP coherence phase histograms. The number in the upper right corner of each plot shows the maximum of circular histogram. The number in the lower right corner shows the number of cells with significant coherence available for phase analysis.

For the cells with significant coherence, the circular mean phase of the significant bins was calculated within the animal-specific beta-band frequency ranges given above. The distributions of the mean phases across the cell populations are shown in Fig. 4 for unit to local LFP coherence (Fig. 4A) and unit to M1 LFP coherence (Fig. 4B). Care should be taken when interpreting histograms with low numbers of cells (e.g. area 2 cells for monkey L), as sampling noise could make these poor representations of the phase distributions of the underlying cell populations. The majority of cells appear to follow a unimodal distribution, with the exception of area 2 cells in monkey L (where the number of cells is too low to draw any clear conclusions), and the area 3a cells in monkey M, which appear to be bimodally distributed, especially in Fig. 4B.

The mean phases (± 95% confidence limits) for unit to local LFP coherence in monkey L were −2.29 ± 0.39 radians for area 3a cells, −0.60 radians for area 2 cells (too few cells to calculate confidence limits) and −1.97 ± 0.20 radians for area 5 cells, with 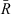
values of 0.37, 0.36 and 0.60, respectively. The corresponding mean phases for monkey M were −1.35 ± 0.51, −2.34 ± 0.18 and −1.57 ± 0.24 radians, with 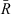
values of 0.24, 0.55 and 0.62, respectively. All of the values of 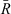
were significantly different from zero (*P* < 0.05, Rayleigh test for uniformity with unspecified mean phase; Fisher, 1993), except for the area 2 cells in monkey L, where only a small dataset was available.

The mean phases for unit to M1 LFP coherence in monkey L were −1.77 ± 0.13 radians for area 3a cells, −0.51 radians for area 2 cells (too few cells to calculate confidence limits) and −2.18 ± 0.19 radians for area 5 cells, with 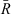
values of 0.46, 0.18 and 0.61, respectively. The corresponding values for monkey M were −1.18 ± 4.05, −2.76 ± 0.17 and −2.04 ± 0.70 radians, with 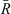
values of 0.04, 0.71 and 0.49, respectively. Most of these 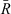
values were significantly different from zero, except for the area 2 cells in monkey L and the area 3a cells in monkey M. For area 3a in monkey M, doubling the phase produced a 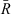
value of 0.37, which was significantly non-zero, confirming the likely bimodal distribution of phase in these cases.

Given the bimodal distribution of the unit to M1 LFP phases for area 3a cells in monkey M, it was of interest to know the distribution of the unit to local LFP phases for the same cells. The cells shown in grey in the unit to local LFP phase plot of Fig. 4A are those that had a positive unit to M1 LFP phase (*n* = 13); these cells also tended to have a positive unit to local LFP phase. We investigated the relationship between unit to local LFP phase and unit to M1 LFP phase for each area and each monkey for those cells that had significant coherence with both local LFP and M1 LFP. We calculated the difference between unit to local LFP phase and unit to M1 LFP phase for each cell. For each monkey–area combination, the mean and 95% confidence limits were calculated for these differences in phase. For most area–monkey combinations, the 95% confidence limits included zero (the differences were not uniformly distributed, Rayleigh test *P* < 0.05, and had means not significantly different from zero, test for specified mean direction, Fisher, 1993). The two exceptions to this were the area 3a cells in monkey L and the area 2 cells in monkey M (these differences were also not uniformly distributed, Rayleigh test *P* < 0.05, but means were significantly different from zero, *P* < 0.05). For the area 3a cells in monkey L, the unit to M1 LFP phases were shifted anticlockwise (mean difference between M1 LFP phase and local phase of −1.18 ± 0.16 radians), and for the area 2 cells in monkey M the unit to M1 LFP phases were shifted slightly clockwise (mean difference of 0.23 ± 0.17 radians).

A number of cells had linear unit to M1 LFP phase–frequency relationships, indicating a fixed delay between neural activity and M1 oscillations. In most cases the slopes were positive, corresponding to the somatosensory and area 5 cells leading the M1 LFP. In monkey M, 6/109 area 3a cells had significant linear regressions of phase on frequency in the beta-band (all positive slopes, mean delay of 21.2 ± 7.8 ms) and 5/90 area 2 cells had significant slopes (all positive, mean delay of 29.5 ± 9.9 ms). No area 5 cells recorded from monkey M had a significant phase–frequency regression. For monkey L, 9/65 area 3a cells (six positive and three negative slopes, mean absolute delay of 30.1 ± 11.0 ms), 6/9 area 2 cells (five positive and one negative slopes; mean absolute delay of 26.8 ± 12.3 ms) and 20/73 area 5 cells (all positive; mean delay of 27.4 ± 8.8 ms) had significant slopes. All values are given as mean ± SD.

### Difference in coherence magnitude between the two monkeys

The unit to M1 LFP coherence magnitude differed by almost 10-fold between the two monkeys from which recordings were available. This could result from a real physiological difference between the two animals. Alternatively, because coherence is a signal : noise measure, the lower coherence in monkey M could result from poorer quality recordings of M1 LFP from the microwire electrodes − for example, due to suboptimal placement or degradation in recording quality due to gliosis around the electrode tip. Because the unit to local LFP coherence results show robust phase locking to local oscillations in both animals (Fig. 2), an explanation based on different recording quality of M1 LFP appears more likely.

**Fig. 5:**
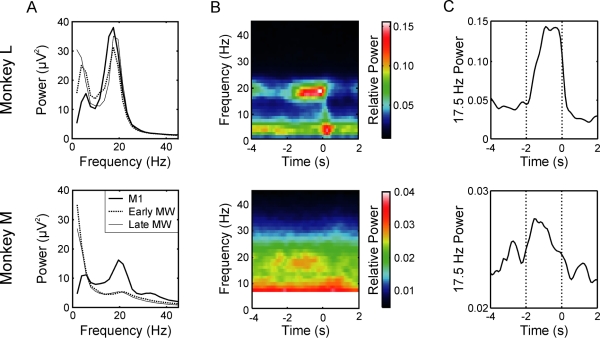
Power spectra of M1 LFP recordings. (A) M1 power spectra calculated for pre-microwire implantation LFP (recorded using acute microelectrodes and the Eckhorn drive), and microwire LFP either early (13 days post-implant for monkey L and 42 days post-implant for monkey M) or late after implantation (76 days post-implant for monkey L and 98 days post-implant for monkey M). Data from each monkey are shown separately. (B) Time-resolved power spectra of M1 microwire LFP for each monkey. Time is shown relative to the end of the task hold period. (C) Plots of 17.5 Hz power vs time for each monkey. The dotted lines indicate the hold period. Time is shown relative to the end of the hold period. M1, primary motor cortex.

To measure the quality of the microwire recordings, we compared the power spectra of the LFPs recorded from M1 using the Eckhorn drive (prior to microwire implantation; recorded from an area with pyramidal tract neurons and clear ICMS effects) with those obtained from the M1 microwires at two different times following implantation. The results are shown in Fig. 5. For monkey L, the three power spectra were similar, suggesting good microwire placement and no noticeable decline in quality over time (Fig. 5A, upper plot). For monkey M there was a clear difference between the acute Eckhorn microelectrode recordings and those from chronic microwires (Fig. 5A, lower plot; thick solid line vs other lines), although the early and late microwire recordings were similar (Fig. 5A, lower plot; thin solid line vs dotted line). The task variation in spectral power of the microwire recordings was calculated using wavelet-based analysis centred on the 2-s hold phase of the task (Fig. 5B). In both animals, there was an increase in 15–25 Hz power during the hold period, although this was greater for monkey M. Figure 5C presents a cross-section through the colour plots of Fig. 5B at 17.5 Hz; this also indicates that oscillations increased during the hold phase for recordings from both animals.

Overall, the data of Fig. 5 confirm that although the implanted M1 microwires successfully recorded beta-band oscillatory activity in both animals, the amplitude of this signal was lower in monkey M. This was probably due to suboptimal initial placement of the electrodes within M1, as no decline in signal quality was seen with time. Such a difference in the signal : noise ratio with which oscillations were recorded almost certainly underlies the differences in unit to M1 LFP coherence observed between the two animals.

### Cells from areas with mainly cutaneous inputs

**Fig. 6:**
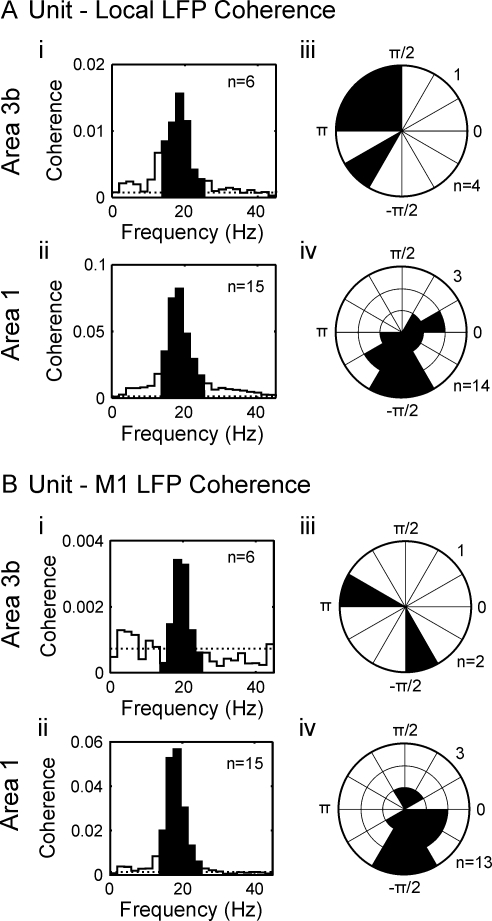
Coherence results for area 3b and area 1 cells recorded from monkey L. (A) Unit to local LFP coherence for area 3b and area 1 cells. (Ai) Average coherence spectrum for area 3b cells. The dotted line shows the significance level. (Aii) Average coherence spectrum for area 1 cells. (Aiii) Phase histogram for area 3b cells. The number in the upper right corner of each plot shows the maximum of circular histogram. The number in the lower right corner shows the number of cells used in the analysis. (Aiv) Phase histogram for area 1 cells. (B) As (A) for unit to M1 LFP coherence. LFP, local field potential; M1, primary motor cortex.

A small number of cells were recorded from area 3b and area 1 in monkey L; this limited dataset was analysed in the same way as described above to allow comparison of proprioceptive and cutaneous areas of S1. Both areas showed clear peaks in their average unit to local LFP coherence spectra (Fig. 6, Ai and ii), and in their average unit to M1 LFP coherence spectra (Fig. 6, Bi and ii). The peak in the area 1 spectrum was particularly large in amplitude (0.057). The peak frequencies were the same as for the other S1 areas in monkey L (17.6 Hz). A high proportion of the cells in area 1 had significant unit to M1 LFP coherence (13/15 cells), compared with only 2/6 cells in area 3b. The distributions of phases are shown in Fig. 6A for unit to local LFP coherence, and in Fig. 6B for unit to M1 LFP coherence. For area 1, the phases were clustered around –π/2 radians for both unit to local LFP coherence (Fig. 6, Aiv), and unit to M1 LFP coherence phase (Fig. 6, Biv). The area 1 population mean phases were −1.33 ± 0.85 and −1.13 ± 0.85 radians for unit to local LFP coherence and unit to M1 LFP coherence, respectively, with 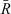
values of 0.51 and 0.56 (in both cases 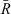
was significantly different from zero, *P* < 0.05, Rayleigh test for uniformity with unspecified mean phase). The small number of cells with significant beta-band coherence in area 3b precluded further analysis of the phase distribution. Cells in these mainly cutaneous areas are also therefore synchronized to motor cortical oscillations in the beta frequency range.

## Discussion

The values reported here for unit to local LFP coherence at ∼20 Hz are broadly consistent with previous studies looking at coherence between M1 pyramidal tract neurons and M1 LFP (Baker *et al.*, 2003), suggesting a similar degree of phase locking to oscillations. For monkey L, the values for unit to M1 LFP coherence across all four S1 areas and area 5 were also consistent with the above study, suggesting that cells in these areas are provided with a similar level of information about the oscillatory state in M1 as single neurons in M1 itself. For monkey M, the values for unit to M1 LFP coherence were much lower. The probable causes for this discrepancy are discussed below.

### Coherence magnitude

In the present data, the size of coherence between single units and M1 LFP varied approximately 10-fold between the two animals (Fig. 2B). This may reflect a genuine difference in the physiology of these individuals, and an underlying difference in the propensity to generate coherent network oscillations. However, the single unit to local LFP coherence in monkey M show that the cells are locking to local oscillations at a comparable magnitude to cells in monkey L (Fig. 2A). Alternatively, it is possible that the difference could be a technical one related to differences in the quality of M1 LFP recordings obtained from the chronically implanted microwires. With such electrodes, a variety of factors including gliosis around the recording tip can cause degradation of the recording over a highly variable time course (Biran *et al.*, 2005; Suner *et al.*, 2005; Griffith & Humphrey, 2006). However, the power spectra of the microwires showed no clear decrease in 20 Hz power between early and late recordings in either monkey (Fig. 5). There was a clear difference between the power spectra of LFPs recorded using the Eckhorn drive (these LFPs were known to be recorded close to pyramidal tract neurons in M1) and the power spectra of LFPs recorded from microwires in monkey M but not in monkey L. This suggests that the major factor in the difference in coherence magnitude was the suboptimal initial placement of microwires in monkey M compared with monkey L.

There was a high correlation between the coherence magnitude of a unit with local LFP, and with M1 LFP (Fig. 3A). Although different units may synchronize to varying degrees with the sensorimotor oscillations, the strength of coupling to oscillations in different areas does not vary independently. This may indicate that oscillations form a global signal whose role is to link together the peri-central cortical areas − possibly engaging them in a common task of sensorimotor integration − rather than a signal of specific local significance.

### Coherence phase

Previous work has reported a phase difference between LFP and cell spiking of around –π/2 radians (Baker *et al.*, 2003; Soteropoulos & Baker, 2006). This probably corresponds to a zero phase lag between the neural activity measured by these two recordings, as extracellular LFP is proportional to the derivative of the transmembrane potential (Hubbard *et al.*, 1969); the derivative operator introduces a phase advance of π/2. In addition, the neural spiking process can introduce a further phase advance between a cell's inputs and its outputs (Matthews, 1997), which could shift phase distributions slightly ahead of –π/2 radians. Phase distributions in the present work often had peaks between –π/2 and –π radians as previously reported for M1 pyramidal tract neuron spikes and M1 LFP (Baker *et al.*, 2003), and the phase dispersion (measured by 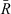) was also similar to values reported previously. The phase of coherence between unit firing and M1 LFP was highly correlated with the phase between unit and local LFP. This is therefore consistent with a zero phase locking between activity in the somatosensory or parietal areas and M1. It is known that there are strong reciprocal cortico-cortical connections between M1 and each of the areas investigated here (Jones *et al.*, 1978; Darian-Smith *et al.*, 1993). Such reciprocal connectivity can lead to zero lag synchronization even in the presence of long conduction delays if the networks include inhibition (Van Vreeswijk *et al.*, 1994; Ernst *et al.*, 1995; Sturm & Konig, 2001). The situation for parieto-motor coherence thus appears similar to the cortico-cerebellar coherence previously reported (Soteropoulos & Baker, 2006).

Anomalously, cells in area 3a in monkey M appeared to have a bimodal unit to M1 LFP coherence phase distribution (Fig. 4B). Some of this bimodality was also present in the unit to local LFP coherence phase distribution (Fig. 4A, grey bins), and there was also high correlation between unit to local LFP and unit to M1 LFP phases (*r*^2^ value of 0.67, slope of 1.02; slope not significantly different from 1). It is known that LFP inverts its phase as an electrode penetrates through the grey matter (Murthy & Fetz, 1996). However, in this case all LFP recordings were made from the same electrodes, which were fixed in place for the duration of the experiments. Phase reversal with penetration depth cannot therefore explain this finding. Ernst *et al.* (1995) showed that reciprocally coupled oscillators could stably synchronize out of phase, as well as in phase. It may be therefore that some cells in area 3a in monkey M showed zero phase, others phase inverted, synchronization with M1 activity. In monkey L, there was a shift of 1.2 radians in the phase distribution between the local LFP coherence and M1 LFP coherence for area 3a. This was not seen for almost all other cell populations − the only exception being area 2 in monkey M, but there the phase shift was very small (0.2 radians). This again emphasizes that area 3a cells may show more complex phase relationships than in the other areas investigated.

### Comparison of different somatosensory areas

Baker *et al.* (2006) found beta frequency coherence between proprioceptive afferents (putative Ia muscle spindles) and forearm muscle activity. By contrast, there was no coherence between muscle activity and an afferent population suggested to relate to cutaneous receptors. In the cortical areas investigated in the present work, we have found no obvious difference in coherence with M1 between regions with mainly cutaneous receptive fields (areas 1 and 3b) and those normally associated with deep receptors and proprioception (areas 3a and 2). Baker *et al.* (2006) suggested from their data that oscillations may have a role mainly in proprioceptive processing; it may be that this needs to be re-evaluated in the light of the present findings. However, in natural situations tactile inputs must be evaluated and interpreted in the light of the limb movements that produced them (‘active touch’, Chapman *et al.*, 1987; Chapman, 1994). Additionally, there is now considerable evidence that proprioception relies partly on cutaneous inputs (Edin & Johansson, 1995; Collins *et al.*, 2005). The very different functions of touch and proprioception cannot therefore be mapped in a one-to-one way to the different receptor classes (cutaneous vs deep). It is perhaps unsurprising that even if a clear separation exists at the receptor level in the extent of oscillatory encoding, this is not seen within the somatosensory cortex.

### Conclusions

These results provide evidence for a close linking of sensory and motor systems via oscillatory synchronization. They further support previous suggestions that this pattern of activity may be important in coordinating the processing of somatosensory information within its motor context ([Bibr b31][Bibr b32]; Witham & Baker, 2007).

## Abbreviations

LFP: local field potential
M1: primary motor cortex
S1: primary somatosensory cortex.
